# Fast-Fed Variability: Insights into Drug Delivery, Molecular Manifestations, and Regulatory Aspects

**DOI:** 10.3390/pharmaceutics14091807

**Published:** 2022-08-27

**Authors:** Nagarjun Rangaraj, Sunitha Sampathi, Vijayabhaskarreddy Junnuthula, Praveen Kolimi, Preethi Mandati, Sagar Narala, Dinesh Nyavanandi, Sathish Dyawanapelly

**Affiliations:** 1 Department of Pharmaceutics, National Institute of Pharmaceutical Education and Research (NIPER), Hyderabad 500037, India; 2 GITAM School of Pharmacy, GITAM Deemed to Be University, Hyderabad 502329, India; 3 Drug Research Program, Faculty of Pharmacy, University of Helsinki, Viikinkaari 5 E, 00790 Helsinki, Finland; 4 Department of Pharmaceutics and Drug Delivery, School of Pharmacy, The University of Mississippi, Oxford, MS 38677, USA; 5 Pharmaceutical Development Services, Thermo Fisher Scientific, Cincinnati, OH 45237, USA; 6 Department of Pharmaceutical Science and Technology, Institute of Chemical Technology, Mumbai 400019, India

**Keywords:** bioavailability, fast-fed variability, food effect, formulation, pH dependent, pharmacokinetics

## Abstract

Among various drug administration routes, oral drug delivery is preferred and is considered patient-friendly; hence, most of the marketed drugs are available as conventional tablets or capsules. In such cases, the administration of drugs with or without food has tremendous importance on the bioavailability of the drugs. The presence of food may increase (positive effect) or decrease (negative effect) the bioavailability of the drug. Such a positive or negative effect is undesirable since it makes dosage estimation difficult in several diseases. This may lead to an increased propensity for adverse effects of drugs when a positive food effect is perceived. However, a negative food effect may lead to therapeutic insufficiency for patients suffering from life-threatening disorders. This review emphasizes the causes of food effects, formulation strategies to overcome the fast-fed variability, and the regulatory aspects of drugs with food effects, which may open new avenues for researchers to design products that may help to eliminate fast-fed variability.

## 1. Introduction

Fast-fed variability is the alteration in the absorption of drugs predominantly due to the presence or absence of food. It is a phenomenon that significantly alters the bioavailability of several drugs [1]. Upon oral administration, drug absorption flux alters depending on a plethora of factors that may increase or decrease absorption, leading to variation in bioavailability [2]. In the case of potent drugs with a low therapeutic index, high fast-fed variability leads to a tremendous increase or decrease in bioavailability, leading to acute/chronic toxicities or therapeutic insufficiencies endangering the patient’s life [3]. Moreover, fast-fed variability poses a great risk for drugs with multiple dosing frequencies, nonlinear pharmacokinetics, greater half-life, etc. In the abovementioned circumstances, designing the dosing regimen will be a challenging task for the physician. Human gastrointestinal physiology is very complex and intricate and aids in the absorption of various nutrients, xenobiotics, chemical moieties, etc., upon oral administration [4]. Highly lipophilic drugs, such as griseofulvin, are more easily absorbed in the fed state than in the fasted state by being solubilized into lipid matrices absorbed from food [5]. Based on the food effect, medications are required to be administered either preprandial or postprandial for better efficacy. This requirement is often problematic in patients with severe disorders treated with multiple medications. These patients often become confused or forget about the dosing instructions, which may lead to incorrect dosing and compromised outcomes [6]. This is a critical issue in the case of geriatrics and pediatrics, wherein patients may forget to take the right medication at the right time [7]. Drugs such as aprepitant [8], bosutinib [9], lurasidone [10], and rivoceranib [11] have been well known to exert fast-fed variability. After decades of development in medicine, this issue has still not been well addressed [12].

**Figure 1:**
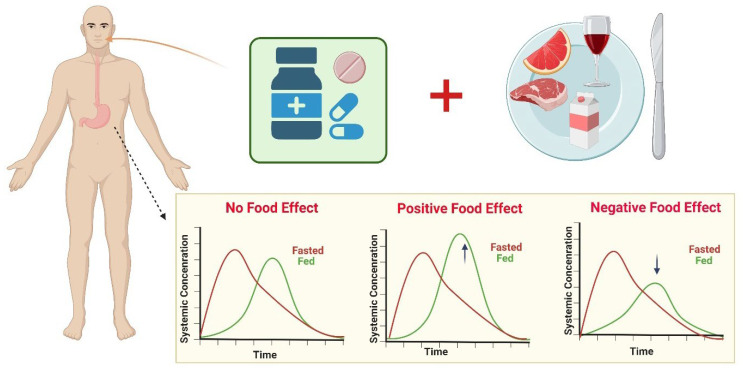
Food role in escalating or diminishing the bioavailability of several drugs; a positive food effect results in an increase in AUC_0–t_ and C_max_, whereas the opposite is seen with a negative food effect. The absence of any kind of effect is known as the neutral food effect. Concept adopted from [15].

The FDA and EMA norms for bioavailability and bioequivalence (BA–BE) studies include taking medication under a fasted state with approximately 240 mL of water after a 10 h overnight fasting period. This will prevent physiological variabilities such as the GI fluid volume, pH, osmolality of gastric components, gastric emptying rate, and the transit time of the drug administered [13]. Food plays a pivotal role in escalating or diminishing the bioavailability of several drugs by mechanisms such as complexation with the drugs, altering the pH-dependent solubility, micellar solubilization, etc. Figure 1 represents the effect of food on pharmacokinetics, categorized into three types: The positive food effect results in an increase in AUC_0–t and_ C_max_ with/without a decrease in T_max,_ whereas the opposite is seen with a negative food effect. The absence of any kind of effect is known as the neutral food effect [14].

Fast-fed variability can also increase intersubjective variability, which in turn increases the need for personalized medications [16]. Therefore, instead of personalized medication, it is advisable to develop a robust formulation with no variability irrespective of the state of dosing. In this regard, this review emphasizes the need to overcome fast-fed variability, factors influencing fast-fed variability, and the aspects with which it can be reduced. It is the need to replace conventional therapy with novel approaches that may help to overcome the adversities of fast-fed variability.

Furthermore, the physiological inter- and intraindividual variability in the fasted and fed conditions factors, strategies to overcome oral drug exposure variability, and experimental methods for measuring or estimating solubility of drugs are discussed exhaustively in the reported review papers [4,15,17,18]. A recently published review discusses pharmaceutical formulation technologies to mitigate the effect of food on drugs and covers preclinical models for forecasting human food [19]. The current review highlights the aspects related to the various formulation-based strategies to overcome the fast-fed variability, patentability, and regulatory aspects, which are not addressed in the previously reported reviews. This may open new avenues for the researchers to design products that may help to mitigate fast-fed variability.

## 2. Factors Influencing Fast-Fed Variability

**Figure 2:**
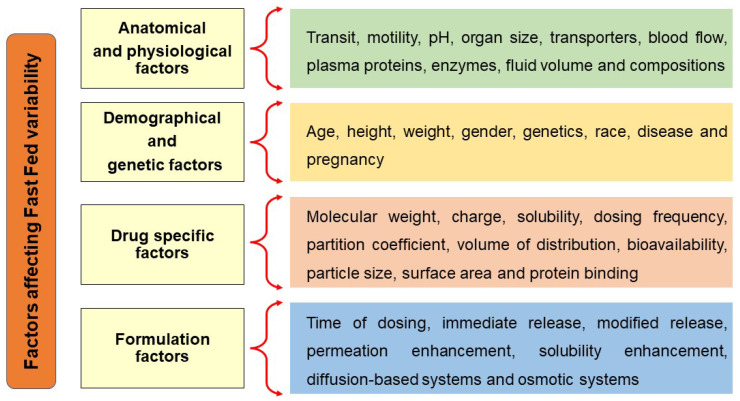
Factors affecting fast-fed variability: anatomical and physiological factors, demographic and genetic factors, drug-related factors, formulation-related factors.

To develop a formulation that can diminish fast-fed variability, it is important to know the factors influencing fast-fed variability, schematically described in Figure 2. A wide variety of factors influence the fast-fed variability; further details are explained below.

### 2.1. Anatomical and Physiological Factors

#### 2.1.1. Gastrointestinal Transit

Gastric emptying is one of the predominant factors influencing fast-fed variability. The gastric emptying rate is governed by the migrating myoelectric complex (MMC) in the fasted state. The MMC cycle takes place specifically between digestion intervals to remove the undigested residue throughout the gastrointestinal tract. In humans, each cycle occurs for 1.5–2 h until the food is consumed. This is divided into four phases. Phase I is the quiescent phase, where no activity occurs up to 40–60 min, to Phase II, where a gradual increment in the frequency of contraction occurs. This is followed by Phase III, where the highest contraction intensity and frequency are reached, and clearance of all undigested chyme occurs, thereby entering the small intestine. This is followed by Phase IV, where the relaxation of gastric muscles occurs [20].

In the fasted state or during intake of liquids, gastric emptying time is less than 30 min, whereas in the fed state, a delay in gastric emptying time is observed, which may be approximately 120 min [21]. Prolongation of gastric emptying time enhances the dissolution of poorly soluble drugs by increasing the time available for solubilization and increases the absorption of drugs from the GIT [22]. Dressman et al. reported a prolongation in the gastric emptying time from 57 min in the fasted state to approximately 102 min in the fed state [23]. The gastric transit time depends on the motility of the GI tract. This motility in turn depends on various factors, such as age, disease conditions, sex, and food content (proteins and fats slow down motility, while carbohydrates enhance motility). Many inter- and intravariations occur that alter GI motility [24]. Other drugs, such as halofantrine and mebendazole. showed increased absorption in the fed state due to prolongation of the gastric emptying time [25]. Few drugs enhance gastric motility owing to their irritancy potential, e.g., cathartics. This reduces the transit time and absorption of several drugs [26,27].

The gastric emptying of solid conventional dosage forms such as tablets and pellets is variable, whereas for liquid solutions, emptying is invariable by the digestive state of the individual. Ogata et al. (1988) reported that small pellets with a size ranging below 1 mm empty from the stomach more rapidly compared to their larger counterparts [28]. However, Clarke et al. reported that pellets of sizes 0.5 and 4.75 mm showed no significant change in gastric emptying time [29]. The small intestine transit time is reported to be constant, i.e., 3–4 h, and a study showed no significant difference between tablets, pellets, and liquids [30]. Feeding and morning awakening have been proven to be major stimuli in provoking colonic motility. The greater fecal bulk is related to a reduced colonic transit time; however, there is no clear justification for the same. Irregular GI motility and variability of bile salts in the different parts of the GI tract may govern drug absorption from the distal parts [31]. Table 1 indicates the length, surface area, and residence time of the GI tract.

#### 2.1.2. Gastric pH

Gastric pH is another major factor contributing to substantial fast-fed variability [35,36]. Dressman et al. explored the alterations in pH attributed to the buffering action mediated by food [37]. The duodenal pH is affected by the fasted or fed state. In the fed state, the pH of the small intestine first falls owing to the acidic chyme from the stomach; however, the fasted state pH is again reached, which is attributed to pancreatic bicarbonate secretion. The pH in the fed state in the duodenum was found to be significantly lower than that in the fasted state, from 4.0–5.4 [22]. Studies show extensive intersubject variability. Among 39 healthy individuals, there was a pH difference of approximately 2 units at the same site [38].

**Figure 3:**
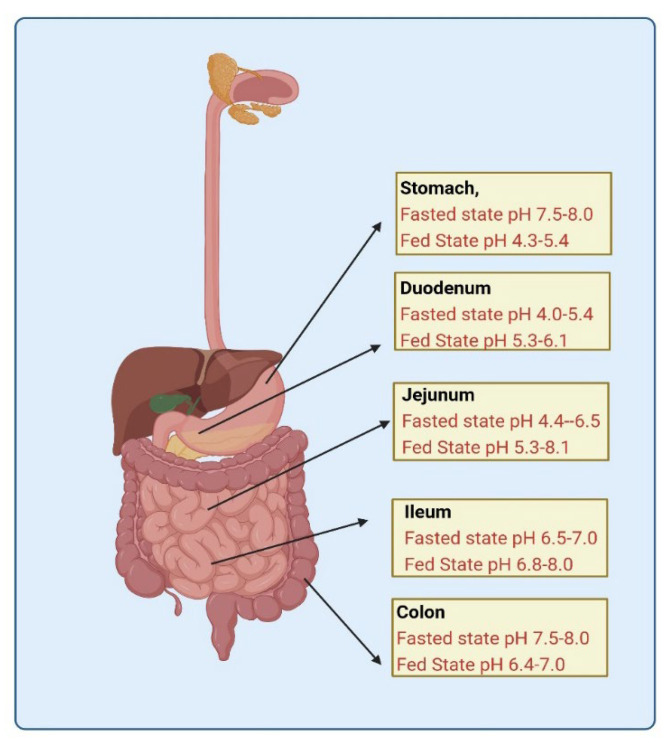
Gastrointestinal tract depicting the diversity in fasted and fed state pH.

**Figure 4:**
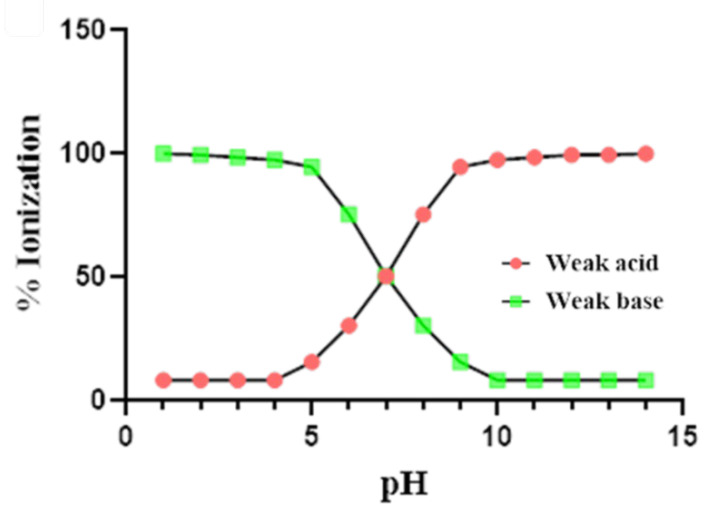
Change in percent ionization of weak acid/base with respect to change in pH.

As shown in Figure 3, the pH of the gastrointestinal tract varies with fast and fed states [39]. Such a variation in fasted and fed state pH alters the solubility of drugs having pH-dependent solubility, thereby modifying the bioavailability of such drugs. As per the pH partition hypothesis, weak bases exhibit high ionization in fasted pH conditions, while weak acids exhibit high ionization in fed pH conditions, as shown in Figure 4.

Since the percentage ionization will determine the solubility of the drug, it can be postulated that weakly acidic molecules in an acidic environment or weakly basic drugs in an alkaline environment will result in decreased or no ionization, leading to an increased percentage of unionized form. In contrast, the presence of food alters the pH, leading to a change in the ionization. In the fed state, a weakly acidic drug is ionized, whereas the weak base remains unionized. This increase in ionization contributes to the absorption flux, leading to increased absorption of the ionized counterpart compared to the unionized form. The unionized drug is also absorbed via passive diffusion with a slower absorption flux. This difference in flux governs the fast-fed variability in drugs with pH-dependent solubility. Most often, an increased unionized form leads to a significant reduction in bioavailability; in such cases, the physician is provoked to administer conventional formulations at high doses, which may subsequently lead to both localized and systemic adverse effects. Additionally, food may increase or decrease the gastro irritancy of some drugs. Increased gastric pH has been reported to reduce the oral bioavailability of several drugs that are acid-soluble, e.g., ketoconazole [40,41], itraconazole [42], dipyridamole [43], indinavir [44], enoxacin [45], cinnarizine [46], and cefpodoxime proxetil [47]. Many drugs exhibit facilitated transport, which becomes the rate-limiting step for their systemic absorption. The number of receptors, as well as transporters, also varies depending on the fasted or fed state, which may change the oral bioavailability of many drugs.

#### 2.1.3. Enzyme Content and Transporters

In the case of oral delivery of peptides, oligonucleotides, and proteins, fast-fed variability is of profound importance. The amounts of peptidases such as pepsin, trypsin, chymotrypsin, etc., vary during the fast and fed states, which may increase or decrease the metabolism of drugs, leading to fluctuations in bioavailability [48]. Subsequently, the content of bile acids for chylomicron uptake of several lipophilic drugs also depends on fast-fed states. Fed state increases bile acid secretion, which increases the amount of chylomicrons that emulsify and promotes the absorption of highly lipophilic drugs via lymphatic uptake [49]. This prevents the first-pass metabolism of drugs, thereby prolonging their circulation time, which increases their duration of action. This is where the drugs with high potency or high dosing frequency need to be carefully monitored to prevent overdosing and concentration-dependent adverse effects of some drugs.

Due to the presence of CYP3A4 enzyme, efflux transporters (ABCB1, MDR1/2/3/4, BCRP, P glycoprotein, MCT1, ENT1/2), influx transporters (OCT 1/2/3, CNT1/2, OCTN1/2, OATP1A2, ASBT, OATP2B1, OATP3A1, PEPT1/2), and variations in the enzyme functions in fasted states can also affect the presystemic metabolism and ultimately the bioavailability of the drugs [2,50]. The presence of food can increase the splanchnic blood circulation, which in turn may increase lymphatic blood flow and may also decrease enzyme concentration in the gut, thereby having a positive effect on bioavailability. Drugs that undergo such a type of enhancement in bioavailability include cyclosporine, midazolam, felodipine, HMG CoA reductase inhibitors, etc. [2]. Cytochrome enzyme activity is modified based on the fasted and fed states. For example, if grapefruit, tomato, or orange juice is included in the diet, they inhibit cytochrome enzymes that prevent the systemic metabolism of several drugs that are CYP substrates, such as propranolol [51] and terfenadine [52]. This leads to an increased residence time and increased bioavailability of the drugs, which may precipitate adverse drug reactions and threaten the well-being of the patient.

#### 2.1.4. Hormonal Changes

During stressful events, various neurotransmitters are released from the brain, thyroid, pituitary gland, and other glands. Few among them are adrenaline, noradrenaline, dopamine, and serotonin, which elicit satiety even in fasted conditions. The body responds in a way as directed by these hormones such that it behaves as it is in the fed state while it is not. This enhances the differences in fasted and fed state bioavailability of drugs, leading to inadequacy or over adequacy to attain desired therapeutic effects [53]. This may in turn increase or decrease the adversity of side effects.

#### 2.1.5. Gastric Fluid Volume and Micellar Solubilization of Lipophilic Drugs

Gastric fluid volume is a vital factor when absorption of a drug is taken into consideration. Each drug molecule has saturation solubility in body fluids. An alteration in the gastric fluid volume may tremendously change the saturation of the drug. During the fasted state, the gastric volume ranges between 13 mL and 72 mL, while during the fed state, the gastric volume ranges between 534 and 839 mL [54]. Gastrointestinal fluid is a multifarious and continuously changing fluid that is indispensable for various rate-limiting steps for dosage forms-disintegration, dispersion, dissolution, and absorption of drugs. It is impacted by the volume of liquid consumed, secretion from the gastric and pancreatic glands, gastrointestinal transit, and the efflux rate of liquids throughout the GI tract.

The flow of the cecum and colon is gradually dampened owing to the reabsorption of water, thereby increasing the bulk phase, which increases the intestinal and colonic transit time. Therefore, drug dissolution is poor due to water inadequacy. Gas bubbles emerging in the colon due to microbial fermentation and degradation reactions may also diminish the connection of the molecules with the mucosa [55]. The contents of the GI fluids alter depending on the physiological stimuli and the rate of secretion. Gastric fluid is a blend of water, hydrochloric acid, electrolytes, and enzymes [56]. Coming to the intestines, the contents of the upper small intestine include chyme, which is passed on from the stomach, along with secretions from different organs, such as the liver, pancreas, and outer layer of the small intestine. The contents are impacted by compartmentalization, mixing configurations, absorption rate, and transit rate [57,58,59].

During the fed state, an enhanced discharge of bile salts occurs compared to the fasted state. Such an increase may be due to the neuronal stimulation of the parasympathetic system sending signals to the liver, pancreas, and gallbladder through neurotransmitters such as dopamine and serotonin. This increases the secretion of bile salts consisting of sodium taurocholate and sodium glycolate [60].

When these bile salts encounter hydrophobic drugs, they align themselves with their hydrophobic surface toward the drug and the hydrophilic end away from the drug. Since their concentration is above the critical micelle concentration, they form micelle-like structures that increase their solubility, reduce their size, and subsequently increase their absorption, leading to a positive food effect. This is mainly prominent in poorly soluble drugs possessing a high partition coefficient. This leads to a significant variation in drug absorption compared to the fasted state [61]. Other drugs, such as halofantrine and mebendazole, showed boosted absorption in the fed state due to micellar solubilization [25].

### 2.2. Demographic and Genetic Factors

Aging is the most important factor that may influence fast-fed variability. Gastric pH, motility, enzyme contents of the body fluids, etc., further depend on the age of the patient. GI transit time increases with age; however, the number of enteric neurons, Cajal cells, and Connexin 43 increases until the adolescent stage and then decreases [62,63]. An aging-associated decrease in peptidase enzymes could lead to decreased metabolism [64]. Fasted and fed state factors become more elaborate during aging, which may be attributed to the variations among interindividual subjects in the aging or injury of their mucosal cell layer and the genetic make-up governing enzyme regulation.

Other predisposing factors affecting fast-fed variability include the dimensional variability of the individual, e.g., height, weight, gender, etc., which influence the drug ADME (absorption, distribution, metabolism, and excretion) properties [65]. The underlying reason for such [66] variability lies within the phenotypic and genotypic variation in the genome of the individuals. This also dictates the basal metabolic rate of the body, which in turn governs the dimensional factors as well as the drug ADME properties. Among the variability in sex, a slower gastric emptying rate is found in women than in men, which may considerably postpone the onset of efficacy of enteric-coated and extended-release forms. The differences in gastric pH may affect the solubility and absorption [67].

Dosage forms with reduced drug release rates may interact with various locations throughout the GI tract, which may contribute to significant variability in terms of the amount of intestinal and hepatic transporters, metabolizing enzymes, hormonal regulation, etc., which may result in tremendous differences in fast and fed state intersex as well as interrace variations [1]. Predisposing epigenetic factors influence enzyme expression and the turnover rate, which dictates the metabolism of labile drugs [68,69]. The population is classified based on the metabolizing rate of enzymes into fast and slow metabolizers. For example, drugs such as caffeine, isoniazid, hydralazine, procainamide, etc., are metabolized by acetylation. The population that has a high acetyl transferase concentration is known as fast acetylators, i.e., Asians, while those having low acetyl transferase concentrations are known as slow acetylators, i.e., African Americans and Caucasians [70]. Such ethnic disparity is also found in other enzymes that contribute to large variability among different races. Diseased states also contribute to fast-fed variability. They are restricted more toward the intraindividual status of the GI tract. Inflammatory conditions such as ulcers, Crohn’s disease, celiac diseases, ulcerative colitis, etc., compromise the permeability and absorption characteristics of the GI tract, which may lead to significant variability. The liver and kidney are the prominent organs for drug metabolism.

Precipitation of organ-related toxicity may further worsen the variability caused by the food effect. This may be due to liver and kidney insufficiency during toxicity, which may impair the functioning of the metabolizing cytochrome and other enzyme families, which may lead to toxicity [71]. It is important to identify the interplay of an individual’s factors that may result in optimal drug therapy [72].

### 2.3. Drug-Specific Factors

The functional groups on the chemical entities play a very important role in contributing to fast-fed variability. These groups contribute to their identifying characteristics, such as pK_a_, partition coefficient, solubility, and molecular weight. Each of these factors may directly or indirectly contribute to fast-fed variability.

#### 2.3.1. Charge

The acid dissociation constant or pK_a_ gives an idea about the nature of the chemical moiety depending on the functional groups present in it. Drugs may be weakly acidic, basic, or neutral. This information can be extrapolated to the degree of its acidic or basic nature, which indicates its pH-dependent solubility potential and the extent to which variations in gastric pH during a fast and fed state affect its bioavailability thereafter. For example, weakly basic drugs such as metformin, raloxifene, epinephrine, etc., exhibit greater solubility in fasted gastric pH compared to fed gastric pH [73]. As mentioned earlier, fasted gastric pH in humans lies around pH 1.7, which increases up to pH 5 in the fed state. For Biopharmaceutical Classification System (BCS)-II drugs, which are weakly basic, a wide variation in bioavailability exists since these drugs have high permeability, and the rate-determining step for absorption is solubility. Since they tend to be more ionized in acidic pH, an increase in solubility by a few fold subsequently increases the absorption flux, resulting in enhanced bioavailability. Similarly, BCS Class II drugs with a weakly acidic nature are better absorbed in the fed state than in the fasted state [74].

#### 2.3.2. Partition Coefficient

The partition coefficient, or at times expressed as “log P”, is a measure of the hydrophilicity/lipophilicity of the drug. This depends on the functional groups present, unsaturation, and molecular weight of the drugs. Generally, but not always, high-molecular-weight drugs are more lipophilic than lower-molecular-weight compounds [75]. This molecular weight governs the absorption mechanism of several drugs through the gastric mucosa, i.e., highly lipophilic drugs are absorbed via passive diffusion, while drugs with low lipophilicity are absorbed via carrier-dependent transport mechanisms. It has been reported that passive diffusion is faster in the fed state. The lipophilic drug moiety is solubilized inside the lipid, followed by micellar solubilization due to the presence of bile salts and chylomicron core formation [76]. These processes make absorption relatively faster than in the fasted state [77].

#### 2.3.3. Molecular Weight

As discussed above, the molecular weight of the chemical moiety governs most of the physical characteristics. Greater molecular weight imparts greater lipophilicity, which may exert a positive food effect on the bioavailability of the molecule. A balance needs to be maintained between hydrophilicity and lipophilicity of the molecule. This balance is described by the Hansen solubility parameter, which is discussed in detail below. To reduce lipophilicity, ionic groups must be attached to the active moieties by prodrugs, complexation, salt forms, etc., which may enhance the solubility irrespective of the food effect, thereby reducing the fast-fed variability [78].

#### 2.3.4. Solubility

Alterations in fast and fed state pH and contents modify the saturation solubility of drugs. As discussed previously, weakly acidic drugs precipitate in acidic pH to a greater extent in a fasted state than in a fed state. When precipitation occurs, there are possibilities of solid-state manipulations, transformations in crystal forms and habits that have even reduced solubility compared to the drug administered [79]. This may be one of the reasons for giving such drugs at high doses to achieve the minimum effective dose.

#### 2.3.5. Particle Size and Surface Area

Particle size and surface area are interrelated factors that affect the solubility of the molecule, resulting in fast-fed variability. A decrease in particle size increases the surface free energy, which subsequently increases the surface area exposed to the surrounding continuous phase [80]. This leads to an enhancement of charged interactions or attraction forces between the drug and the surrounding media, thereby enhancing the dissolution rate of the drug. A significant increase in magnitude occurs in the case of drugs with high pH-dependent solubility, wherein this property of the drug is increased by severalfold with an increase in surface area. Drug entities with a reduced particle size have an enhanced dissolution rate, along with an enhanced absorption flux compared to the particles with an increased size [81]. During the fed state, where pH increases due to the presence of food, lipophilic drugs with a reduced particle size show an enhanced dissolution rate and absorption owing to their size, surface area, micellar solubilization, and other mechanisms [22]. Drug nanonization follows a similar mechanism to drug micronization with only enhanced attributes. Both approaches can be used to overcome fast-fed variability; however, the flux of the drug across the membrane becomes greater in fasted as well as fed states for nanonized systems compared to micronized systems irrespective of pH-dependent solubility of the drugs. Jinno et al. reported no statistically significant difference in the fasted and fed state absorption of Cilostazol nanocrystal^®^ technology compared to jet-milled and hammer-milled techniques owing to reduced particle size and subsequent increase in dissolution rate far more superior to other techniques. Pharmacokinetic studies showed statistically insignificant differences in the bioavailability of the drug in fasted and fed states [82].

#### 2.3.6. Pharmacokinetic Factors

Various pharmacokinetic factors may play a key role in worsening the effects of fast-fed variability. The half-life, volume of distribution, plasma protein binding, etc., may contribute to adverse drug-related toxicities if its absorption is increased when the dose is consumed other than the directed indication. An increased bioavailability of high-protein-bound drug was observed in the patient because of the food intake. As a result of the longer residence period in the body, such patients need to be watched carefully to prevent serious toxicities [83]. This may increase the propensity of severe side effects if the dosing regimen is continued for a prolonged duration. Dosing frequency and the volume of distribution govern the adversity and the location of potential toxicity, which needs to be anticipated by the physician.

### 2.4. Formulation-Related Factors

**Figure 5:**
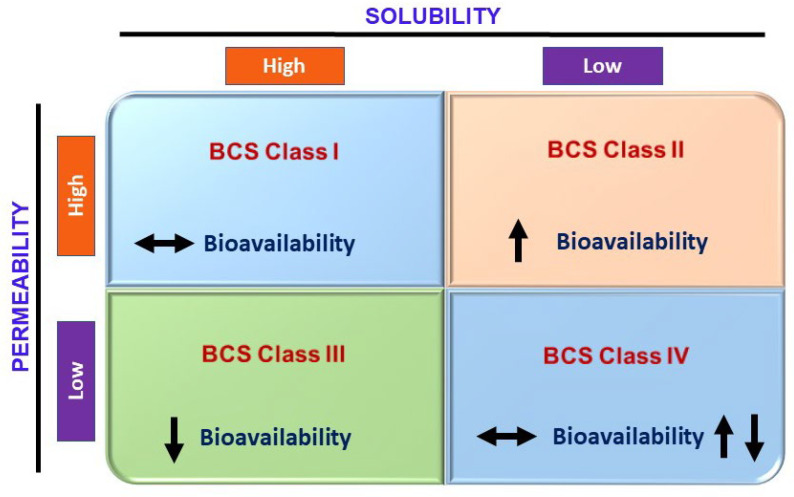
Effects of food on changes in bioavailability as a function of BCS class, concept adopted from [87].

Formulation-related factors are the pharmaceutical factors that play an important role in contributing to fast-fed variability. These predominantly include the release rate kinetics of a drug from the dosage form. The extent of a drug undergoing fast-fed variability can be altered with the help of kinetics and the mechanism of drug release, i.e., pH-responsive, osmotic, diffusion-controlled, erosion control [84]. A significant food effect is observed in immediate release dosage forms if the drug is susceptible to the food effect [85]. Efficient control of formulation-related factors can help to effectively reduce the effects of food on changes in bioavailability variability. The rate-determining step could be drug dissolution, which is influenced by pH, micellar solubilization, and other factors, i.e., BCS classes II and IV. It is well known that food can change the solubility and permeability of drugs. Wu and Benet predicted the influence of food on solubility and permeability as a function of BCS [86]. Figure 5 summarizes the effects of food on bioavailability changes.

Therefore, formulation approaches implying amelioration of the solubility and dissolution rate independent of pH are desirable, i.e., lipidic emulsifying systems, solid dispersions, cyclodextrin complexations, etc. Since they suffer from solubility challenges, the food effect may be highly pronounced if they are incorporated into immediate release dosage forms. For BCS class I drugs, immediate release dosage forms are preferable. For class III drugs, the use of prodrugs and lipidic systems may help improve the partition coefficient of these hydrophilic drugs, enhancing drug absorption in the fed state owing to micellization and other mechanisms [88]. Osmotic systems may also control drug release and cause pH- and food-independent drug release. For BCS, class IV drugs suffer from solubility and permeability challenges.

## 3. Formulation Strategies to Overcome Fast-Fed Variability

**Figure 6:**
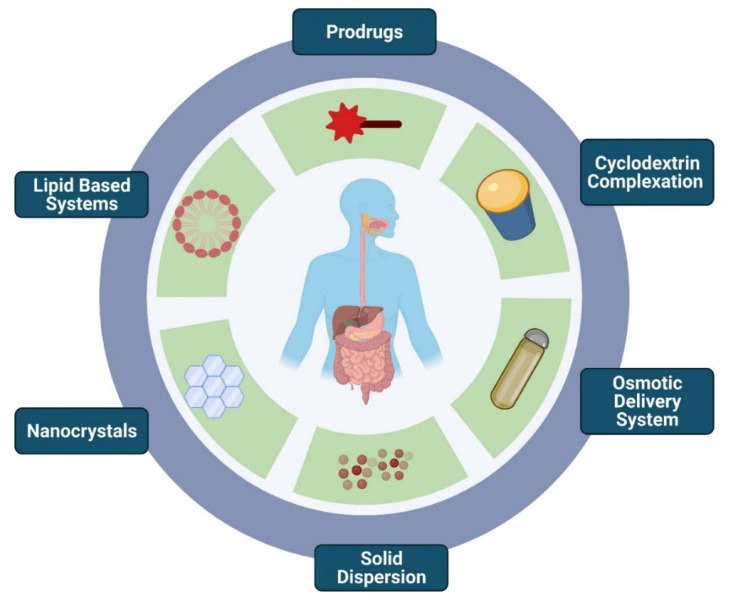
Formulation strategies to overcome fast-fed variability: prodrugs, cyclodextrins, osmotic delivery systems, amorphous solid dispersions, nanocrystal technology, and lipid-based systems.

As discussed previously, one can witness the apprehensive effects of the factors influencing fast-fed variability. Figure 6 enumerates various formulation strategies to overcome fast-fed variability. To overcome these obstacles, a series of methods were attempted.

### 3.1. Prodrugs

The prodrug approach aims to form covalent bonds between functional groups, such as hydroxyl, amide, acid, ester, etc., of the drug with different moieties, which alter the partition coefficient and solubility in such a way that the final product formed is absorbed irrespective of the fasted and fed state [89,90]. Once absorbed, the prodrug undergoes metabolism and releases an active moiety into the circulation. Ximelagatran, a prodrug of melagatran used for the treatment of platelet aggregation, was formulated by Astra Zeneca. This drug is poorly bioavailable due to its high hydrophilicity, and its bioavailability is greatly affected by food. Ximelagatran contains a carboxylic acid group that is transformed into an ester group, while the imidine group is hydroxylated to decrease the basicity of the molecule. It is unionized at alkaline pH, making it 170 times more lipophilic and 80 times more permeable than Melagatran [91]. Fosamprenavir is a prodrug of amprenavir used as an HIV protease inhibitor. Commercial Fosamprenavir (Telzir^®^) tablets were administered to five volunteers in the fasted and fed state. The results indicated that intake of food delayed the gastric dissolution of the drug, leading to delayed absorption [92]. The bioavailability of gabapentin is higher than that of its ester prodrug. Horizant^®^, consisting of gabapentin enacarbil, needs to be administered in the fed state, while Neurontin^®^, which contains gabapentin in its pure form, does not show fast-fed variability [93]. Lee et al. reported the use of trypsin in binding with LB30870, a new direct thrombin inhibitor, to reduce its negative food effect [94]. Azilarsartan medoxomil, a BCS class IV drug, is hydrolyzed to release the active moiety azilsartan by esterase in the gastrointestinal tract, which does not affect its bioavailability [95]. Other prodrugs whose absorption is unaffected by food include enalaprilat [96], fesoterodine [97], and fludarabine phosphate [98].

### 3.2. Cyclodextrin Complexation

Cyclodextrin complexes are widely used to enhance the solubility and permeability of several BCS Class II and IV drugs [99]. Cyclodextrins are known to incorporate hydrophobic drugs into their inner cavity, while their outer hydrophilic surface surrounds the outer aqueous environment. This approach also helps to overcome the pH-dependent solubility of weak acids or bases since the reduced solubility at pH at which the drug is unionized is compensated by complexation with cyclodextrin [100].

Itraconazole, a BCS class II drug, is known to show a positive food effect, i.e., its bioavailability increases significantly in the presence of food [101]. Velde et al. reported hydroxy propyl-β-cyclodextrin complexes of itraconazole to investigate its effect on fast-fed variability [102]. The cyclodextrin complexes reported an increase in bioavailability in healthy volunteers of itraconazole in a fasted state compared to the fed state owing to its increased solubility and dissolution rate, thereby reducing the difference between the bioavailability among fasted and fed state. Sporanox^®^, a product from Janssen Cilag, was marketed as an oral hydroxy propyl-β-cyclodextrin inclusion complex solution. It showed an enhanced bioavailability compared to conventional itraconazole capsules irrespective of fasted or fed conditions [102].

**Figure 7:**
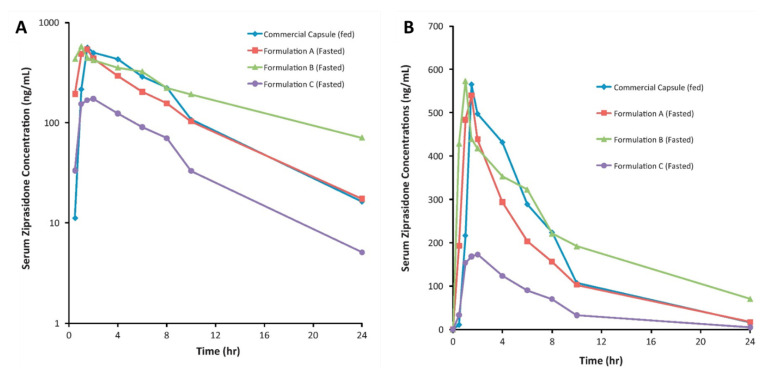
(**A**) Semilog plot) and (**B**) linear plot of mean dose-normalized serum ziprasidone concentration versus time after dosing of capsule in the fed state and the test formulations in the fasted state. Reprinted with permission from Ref. [103]. Copyright 2012, Elsevier.

Thombre et al. formulated amorphous, nanocrystalline, and crystalline ziprasidone formulations, which subsequently improved solubility as well as bioavailability and eradicated the fast-fed variability. The amorphous complex and the nanosuspension ziprasidone formulations displayed enhanced absorption in fasted beagle dogs compared to Geodon^®^ capsules. These solubilization technologies have the potential to reduce the food effect in humans, as shown in Figure 7 [103].

Similarly, Wang reported sulfobutyl ether (SBE) cyclodextrin complexes of amiodarone hydrochloride (AME), a drug with high pH-dependent solubility toward acidic pH. The cumulative dissolution of the cyclodextrin complex showed greater than 85% in vitro drug release in water, pH 4.5 acetate buffer, and 0.1 N HCl buffer solutions. The pharmacokinetic studies demonstrated no significant difference in the absorption of the AMI-SBE-β-CD inclusion in both fast and fed states [104]. Recently, an inclusion complex of lurasidone hydrochloride with SBE cyclodextrin was developed to reduce the food effect, and the authors demonstrated the improvement of bioavailability and showed elimination of the food effect [105].

### 3.3. Osmotic Delivery System

An osmotic delivery system could be ideal for overcoming fast-fed variability since the coat is merely permeable to water and a zero-order release is obtained irrespective of GI conditions. This may help in reducing the food effect of such drugs compared to their conventional counterparts owing to the independence of drug release on pH or any other factor [106]. Modi and coworkers investigated the effect of a high-fat meal on the pharmacokinetics of OROS^®^ (osmotic controlled-release formulation of methylphenidate HCl) in healthy subjects [107]. They reported AUC_0-∞_ values of 1857 ng.h/mL for fasted subjects and 1872 ng.h/mL for fed subjects and C_max_ values of 112.6 ng/mL for fasted subjects and 124.9 ng/mL for fed subjects. They concluded that the nonexistence of food affects the absorption of methylphenidate in patients subjected to no food as well as a high-fat meal [107].

Yanfei et al. developed ziprasidone solid dispersion-loaded osmotic pump tablets to reduce the food effect of ziprasidone. They reported a fasted state and fed state C_max_ of 294.3 ± 74.5 ng/mL and 311.7 ± 64.5 ng/mL, respectively, with a fasted state AUC_0-∞_ of 3974 ± 314.5 ng.h/mL and a fed state AUC of 3812 ± 314.5 ng.h/mL. The food effect was eradicated by a combination of solubility enhancement of solid dispersion and the zero-order release of osmotic pump tablets [108].

### 3.4. Amorphous Solid Dispersion

Amorphous solid dispersions (ASDs) are well-known and popularly used techniques in the pharmaceutical industries today to convert crystalline drugs into amorphous materials via solid-state manipulations [109]. Lipinski et al. demonstrated that solute solubility is dependent on the crystal packing energy, cavitation energy (energy required to shift water and create a cavity into the solute molecular arrangement), and solvation energy (energy released due to favorable interactions between the solvent and solute) [110]. This approach enhances the solubility by fewfold since it increases the surface free energy and entropy and breaks the molecular packing. ASDs also show a spring parachute effect since they are a part of supersaturated systems. The spring parachute effect, a characteristic of cocrystals and ASD, is an effect where a burst increase in the drug dissolution rate occurs, which is maintained over time. Once almost all the drug has been released for a particular duration of time, the solubility then decreases. The burst release effect corresponds to a spring, and its maintenance and a gradual decrease in the dissolution rate with time are described as a parachute. The amorphous form of the drug is more soluble than the crystalline form at different pH ranges, which improves its bioavailability irrespective of the presence or absence of food [111]. This approach is suitable for BCS Class II drugs whose bioavailability is hindered by limited solubility. For drugs that have pH-dependent solubility and exhibit variations in absorption in fasted and fed states, this approach can be used by taking advantage of the amorphous form and the hydrophilic carrier to improve the dissolution rate of the drug. Hot-melt extrusion technology was employed to develop an amorphous solid dispersion of a fixed-dose combination of lopinavir and ritonavir for AIDS treatment, commercially known as Kaletra^®^ (AbbVie Inc., North Chicago, IL, USA) [112,113]. Before ASD, it was originally dispensed as a soft gelatin capsule with lipidic excipients with a high capsule burden and dosing frequency of four capsules per day. Solid dispersion of the combination reduced the dosing frequency to two tablets and successfully eradicated fast-fed variability [114]. AstraZeneca developed ASD-based formulations with olaparib to improve solubility, which improved bioavailability as well as drug loading along with a significant reduction in the food effect [115]. Ziprasidone solid dispersion resulted in significant improvement in solubility and bioavailability, thereby abolishing the variability concerning an absence of statistically significant differences in C_max_ and AUC in fasted and fed states compared to the commercial Zeldox^®^ formulation [116]. Othman et al. prepared melt-extruded and spray-dried solid dispersions for the drug ABT-102. They reported an increased oral bioavailability compared to the plain drug and similar C_max_ and AUC for fasted and fed state (melt extruded solid dispersion–fasted state C_max_ 9.4 ± 2.3 ng/mL and fed state C_max_ 9.4 ± 3.2 ng/mL; AUC_fasted_ 109 ± 35 ng.h/mL and AUC_fed_ 112 ± 35 ng.h/mL) and for spray-dried solid dispersion (fasted C_max_ 8.6 ± 2.7 ng/mL and fed state C_max_ 9.2 ± 3.0 ng/mL; AUC_fasted_ 89 ± 37 ng.h/mL and AUC_fed_ 107 ± 53 ng.h/mL) [117]. Table 2 lists the marketed formulations that have successfully overcome fast-fed variability.

### 3.5. Nanocrystal Technology

Nanotechnology offers a wide range of possibilities in improving the therapeutic potential of various molecules in different indications [118,119,120,121,122,123,124,125,126,127,128]. Furthermore, nanonized delivery systems have the potential to overcome fast-fed variability. Nanocrystals or nanosuspension technology is one such technology that uses either “top down” or “bottom up” approaches for particle size reduction using antisolvent addition, supercritical antisolvent techniques, and sonoprecipitation methods, respectively [129,130]. Nanosuspensions enhance the dissolution and permeability characteristics of BCS Class II and BCS Class IV drugs owing to their nanometric size range, greater surface area and surface energy, amorphization taking place during the process of size reduction, etc., leading to an increased solubility and dissolution rate [10,103,131]. Rangaraj et al. developed an ibrutinib nanosuspension and depicted a reduction in fast-fed variability via simulated gastric fluids and in vivo pharmacokinetic studies [131]. Two marketed nanocrystal preparations of fenofibrate-Tricor^®^ and Triglide^®^ were compared for their absorption with microcoated fenofibrate tablets in the fasted and fed state. They revealed similar absorption characteristics in the fed state, while absorption from the nanocrystal tablet was enhanced in the fasted state, which led to the elimination of fast-fed variability [132,133].

**Figure 8:**
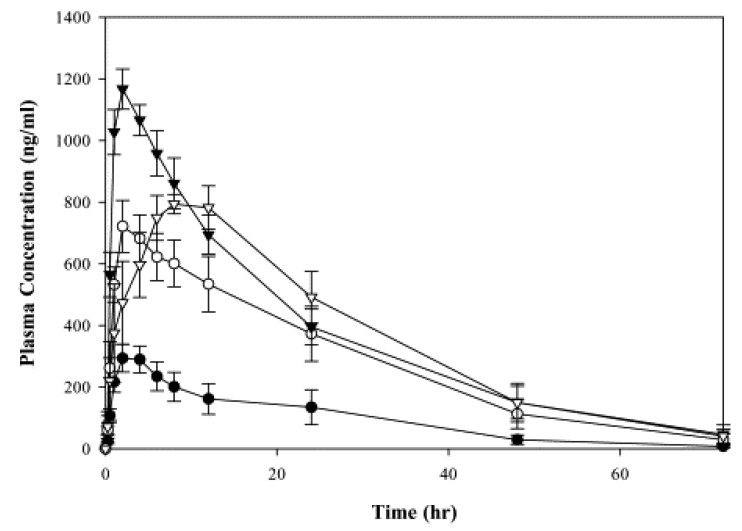
Comparison of mean plasma concentrations of MK-0869 after oral dosing in beagle dogs (*n* = 5) of suspension (●, fasted; o, fed) with NanoCrystal^®^ dispersion formulation (▾, fasted; ▿, fed). Reprinted with permission from Ref. [134]. Copyright 2004, Elsevier.

Aprepitant (MK-0869), a BCS class IV drug, uses Nano Crystal^®^ technology to improve drug dissolution in the fasted state [134]. The formulation was found to eradicate the positive food effect observed with tablet formulations. There was an enhancement in both AUC (3.2-fold) and C_max_ (2.3-fold) observed in the fed-state beagle dogs, as shown in Figure 8 [134]. Megestrol acetate, a steroidal progestin, displays a positive food effect when incorporated as an oral suspension. Megace ES^®^ nanocrystals were developed, which demonstrated a reduction in fast-fed variability [135]. Jinno et al. prepared a spray-dried nanocrystalline suspension of cilostazol that diminished the positive food effect seen with the micronized formulations (C_max_ fed/fast = 0.91 ± 0.13 ng/mL AUC fed/fasted = 0.76 ± 0.04 ng.h/mL and mean residence time fed/fast = 0.95 ± 0.13 h, respectively). This was attributed to improved dissolution, which increased the absorption flux [82].

### 3.6. Lipid-Based Systems

Many lipid-based formulations have the potential to reduce fast-fed variability [136]. Among these formulations, self-micro/nanoemulsifying delivery systems (SMEDDSs/SNEDDSs) are one of the most efficient formulations and can be used to overcome the food effect [137,138]. Lipid-based formulations report an increase in bioavailability among both fasted and fed states, which may be due to nanometric size in the fasted state and enhanced absorption via micellar solubilization with the help of chylomicrons during the fed state [139]. Poorly hydrophilic drugs are solubilized into the lipid matrix, which, with the help of emulsifiers when encountering water in gastric fluid, forms a nanoemulsion. The lipid globules formed during the o/w emulsion process promote the secretion of bile salts in the fasted state. The secreted bile salts form mixed micelles with the oil globules in which the drug is dispersed and thereby aid in drug absorption [140]. Since the mixed micelles are formed in both fasted and fed states, a significant difference in the absorption of drugs does not exist. Additionally, these lipids may be absorbed via lymphatic transport with the help of chylomicrons, which also helps prevent hepatic first-pass metabolism thereafter [141,142].

SNEDDSs are more stable than nanoemulsions since the nanoemulsion is formed in situ. Porter et al. suggested three possible mechanisms for drug absorption: (1) alteration of the composition and character of the intestinal secretion, (2) intestinal lymphatic drug transport, and (3) enterocyte-based transport processes [143]. Lurasidone HCl, a BCS class II drug, shows a positive food effect attributed to delayed gastric emptying in the fed state, prolonging the time accessible for drug solubilization. Oral administration of lurasidone into a phospholipid-based self-nanoemulsifying self-nanosuspension (p-SNESNS) system demonstrated a reduction in fast-fed variability; this improvement is due to enhanced solubility [144]. Another study included coadministration of Sepan^®^, a marketed conventional cinnarizine tablet, along with placebo SNEDDS to investigate the role of SNEDDS in emulsifying cinnarizine from the tablet. Failure to reduce fast-fed variability on cinnarizine bioavailability to a statistically significant level was observed [145].

**Figure 9:**
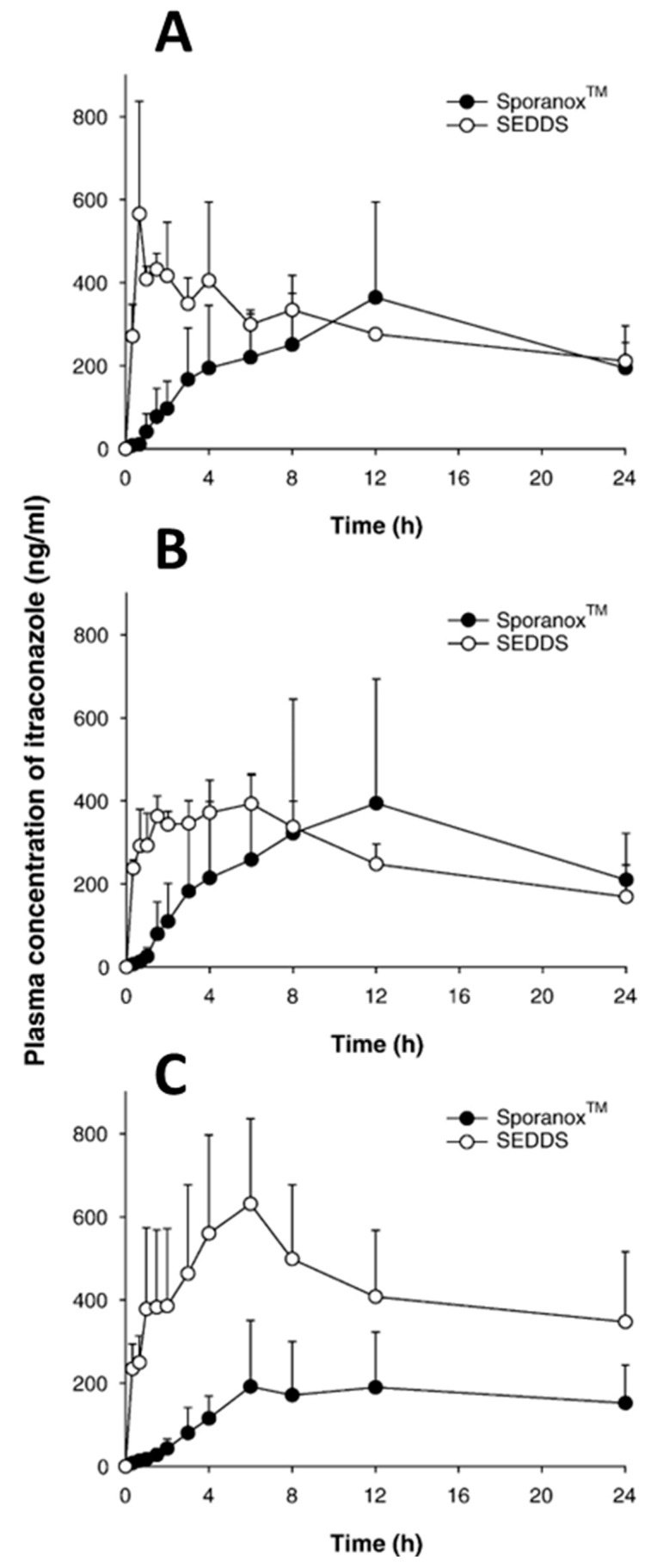
Plasma concentration of itraconazole versus time after oral dosing of the Sporanox^®^ capsule and itraconazole-based SEDDS. Fasted overnight (**A**), normal diet (**B**), and lipidic diet for 1 day (**C**). Reprinted with permission from Ref. [148]. Copyright 2006, Elsevier.

Miao et al. reported an elimination of fast-fed variability for lurasidone-loaded SNEDDSs in beagle dogs with similar C_max_ and AUC values in fasted and fed states [146]. A study was performed to investigate the efficacy of ziprasidone-loaded SNEDDSs in sustained-release pellets to improve oral bioavailability and mitigate the food effect on ziprasidone absorption. They found a statistically insignificant difference between the increase in bioavailability in the fed and fasted states, 1.578- and 1.501-fold, respectively, compared to the Zeldox^®^ capsule. The eliminated fast-fed variability was attributed to enhanced lymphatic transport and increased dissolution rate due to increased surface area owing to reduced droplet size [147]. Another study was employed to examine the differences in gastrointestinal absorption between itraconazole-based SMEDDSs (equivalent to 15 mg of drug/kg of body weight) and conventional Sporanox^®^ capsules in Sprague Dawley rats. Due to its enhanced solubility and in vitro dissolution of itraconazole in SMEDDS preconcentrates, an increased oral bioavailability of itraconazole was observed in different dietary conditions. This study concluded that the SMEDDS formulation showed increased absorption in rats irrespective of fasted and fed states, as shown in Figure 9 [148].

**Figure 10:**
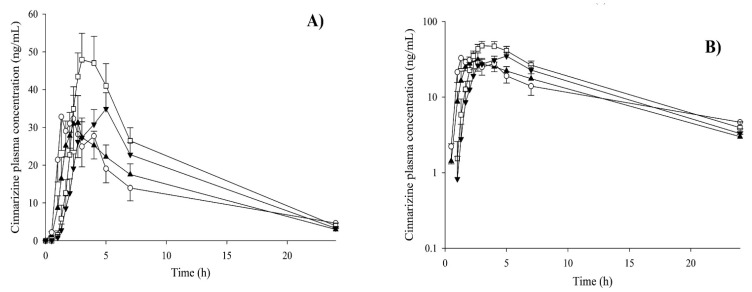
(**A**) Normal scale, (**B**) log scale of mean plasma concentration of cinnarizine versus time curves in both fast and fed conditions; tablets in fasted state (ο); tablets in fed state (□); tablets + SNEDDS in fasted state (▲) and tablets + SNEDDS in fed state (▼) (*n* = 10). Reproduced with permission from Ref. [145]. Copyright 2016, Elsevier.

Dening et al. reported a significant reduction in the variation of ziprasidone solubilization between the fasted and fed states owing to its incorporation into SNEDDS. The incorporation of ziprasidone into SNEDDS eliminated the dissolution step required for drug absorption, thereby eliminating the effect of diet [149]. Apart from the fed state, self-emulsifying delivery systems could be used to abolish the impact of certain food contents on drug absorption. The oral bioavailability of itraconazole in SEDDS in the fasted state was comparable to that in the fed normal food as well as fed lipidic food post-administration to male Sprague Dawley rats [148]. In another study, commercial conventional cinnarizine tablets (Sepan^®^) were administered to 10 human volunteers in both fasted and fed states, with and without coadministration of a placebo SNEDDS capsule [145]. The SNEDDS formulation study investigated the pharmacokinetic difference between the conventional tablet of cinnarizine vs. the SNEDDS formulation, which resulted in a reduction in the food effect and increased absorption (Figure 10). After co-administration of SNEDDS with tablets, the pharmacokinetic study indicated an increased bioavailability of cinnarizine in the fasted state and reduced bioavailability of cinnarizine in the food effect, as shown in Figure 10 [145]. Table 3 indicates various strategies along with reported pharmacokinetic study data for formulations reducing food effects.

## 4. Interplay of Different Molecular Properties Contributing to Fast-Fed Variability

The concepts of “like dissolves like” or often described as “like seeks like” given by Hansen, Hildebrand, and Scott seem to play a key role in pH-dependent solubility, which can be extrapolated to pharmacokinetic fast-fed variability. As per Hansen’s solubility parameter, solubility depends on the sum of partial cohesive energies (dispersive forces, hydrogen bonding, and permanent dipoles) divided by its molar volume [150]. The strategies discussed above use this principle to reduce the food effect. For instance, cyclodextrins, solid dispersions, nanocrystals, etc., depend on these partial cohesive energies (mainly hydrogen bonding and dipolar interaction with the hydrophilic polymer), thereby increasing the solubility of the drug irrespective of the variations in the gastrointestinal tract pH [19]. Prodrugs devoid of fast-fed variability aim to balance the partition coefficient of drugs during absorption, i.e., highly hydrophilic drugs may require lipophilic moieties to impart appropriate log *p* values to be absorbed in the present as well as the absence of food. Lipidic emulsifying systems disperse the drug into their nanosized oil droplets, which increases the surface area for increased dissolution and may also participate in bile-salt-aided mixed micelle formation and lymphatic uptake. Out of a large array of drugs, some show variability in absorption, which is measured in terms of fasted and fed state pharmacokinetics by taking into consideration the area under the curve (AUC), which gives an idea about the change in bioavailability and maximum plasma concentration (C_max_) concerning fasted and fed state.

From Table 4, it is very much understandable that most of the drugs undergoing fast-fed variability in vivo may be due to increased pH-dependent solubility. Of these drugs, isotretinoin, fenofibrate, and elbasvir/grazoprevir show a positive food effect, which may be attributed to the pH-dependent solubility in the increased fed state pH as well as their high lipophilicity compared to the fasted state. Other drugs, such as cefuroxime axetil and olaparib, possess reduced lipophilicity and have greater solubility in the acidic pH of the fasted state but surprisingly showed a positive food effect, i.e., AUC_fed_/AUC_fast_ greater than 1, which may be due to increased residence time during the fed state compared to the fasted state. Drugs such as vemurafenib, sirolimus, netupitant, progesterone, and aprepitant possess high lipophilicity and pH-dependent solubility in acidic pH; however, they show a positive food effect, which may be due to the chylomicron-assisted emulsification of the bile salts during the fed state contributing to enhanced bioavailability compared to the fasted state. Drugs such as rosuvastatin calcium and topotecan HCl show reduced lipophilicity and a negative food effect, which may be due to the formation of complexes with the food contents, thereby reducing their bioavailability when taken with food [151]. The remaining drugs, including tacrolimus, ritonavir, everolimus, quinapril HCl, amprenavir, nifedipine, lubiprostone, paricalcitol, tafamidis meglumine, and itraconazole, show a negative food effect even with increased lipophilicity, which may be due to their increased solubility in the acidic pH of the fasted state. We can conclude that pH-dependent solubility may play a prime role in contributing to the fast-fed variability of several drugs.

## 5. Regulatory Aspects

Food plays a significant role in variations in intra- and interindividual bioavailability. Factors influencing such variations have been explained previously. As per the study carried out by the USFDA, guidance for the pharmaceutical industry was documented and fabricated in December 2002 for directing bioavailability and bioequivalence (BA–BE) studies. The protocol stated an improved standardization being achieved during the trials, leading to a better knowledge of the observed mechanisms leading to fast-fed variability as well as the effects of the same. Recently, the European Medicines Agency (EMA) reformed its norms, taking into consideration the recommendations of the FDA. Today’s FDA and EMA norms and guidelines demand the administration of a large calorific intake of approximately 850–1000 kcal to check the fast-fed variability in the oral bioavailability of the drugs under study. This meal should derive approximately 150 kcal of protein, 250 kcal of carbohydrate, and approximately 500–600 kcal of fats. FDA and EMA norms together consist of a protocol for BABE studies specifically for the diet of the subjects participating in the study to maintain diet uniformity among individuals [152,153].

The drug product required for testing was given 30 min postmeal consumption along with 240 mL of water. The assessment of fast-fed variability is evaluated on the ratios of AUC and C_max_ acquired after drug incorporation under fasted and fed states, respectively. The acceptance criterion may lie between 80–125% but may be wider or narrower depending on the bioavailability of the drug. There is a wide opportunity for the ever-growing pharma industry to extend the patent terms of their new molecular entities as well as marketed drugs and formulations by exploiting the benefits offered by the previously discussed pharmaceutical opportunities to diminish fast-fed variability. This may also help the applicant seek approval of their products via the ANDA 505(b)(2) pathway, wherein a formulation may be claimed superior compared to the innovator product by being able to reduce the fast-fed variability, which is a whole new domain waiting to be explored. This not only ameliorates patient compliance but also reduces the adverse effects of drugs due to positive or negative food effects. To overcome patenting obstacles, the formulation data can be projected in such a way as to prevent patent infringement from other inventors as well as self-patented entities. Table 5 depicts the number of patents applied by various inventors to date successfully without infringing the preexisting patents by overcoming the fast-fed variability of several drugs by different formulation approaches. This not only contributes to society but also helps generate revenues for the innovator company to encourage them to promote further research and development to bring new molecules into the market.

## 6. Concluding Remarks

This review provides a critical evaluation of the various fast-fed variable-causing elements, GIT consideration insights, and a thorough note on formulation techniques to overcome the fast-fed challenges. Despite the plethora of research, a considerable translational gap hinders such formulations from becoming commercially viable. To anticipate the effects of food in vivo more accurately, future research should concentrate on improving dynamic in vitro models that analyze dissolution, solubilization, and permeation concurrently. Strategies such as prodrug approach, cyclodextrin complexation, osmotic delivery system, solid dispersions, nanocrystal technology, and SNEDDS provide therapeutic benefit by mitigating the fast-fed variability. Several aspects drive the decision on final formulation, such as cost–benefit analysis, ease of administration, and molecule properties. Since the oral route is the simplest and most used route of delivery it is important to consider the strategy that is not burdensome to the patients. Nanotechnological approaches sometimes offer distinctive advantages over other traditional methods; however, the equipment used and scalability add up cost on the final formulation. In addition, the regulatory guidelines vary a great extent if one of the formulation components is in the nano range, i.e., at least one component of final formulation is in the range of 1–100 nm. This could be one reason for predominant usage of traditional methods. In a report by Kola and Landis, it was evident that there was substantial reduction in attrition rate due to PK/bioavailability between 1991 to 2000 [180]. This was attributed majorly to the improved formulation strategies at early stage of development. A further analysis is much needed for the last decade. Additionally, it would be beneficial to have a framework to help with formulation strategy selection to counteract the impacts of food. However, proper medication reformulation to lessen the pharmacological food impact is very helpful in lowering pharmacokinetic variability, allowing uniform drug administration regardless of ambient factors. Regulatory aspects regarding the food effect and its importance in patents and ANDA applications were discussed. We conclude that various approaches described in the review are useful in the formulation process and can be used to eliminate the fast-fed variability of several drugs.

## Figures and Tables

**Table 1:** Characteristics of gastrointestinal tracts, data references [32,33,34].

S. NO.	Gastrointestinal Tract	Length (m)	Surface Area (m^2^)	Residence Time
1.	Esophagus	0.3	0.02	30 s
2.	Stomach	0.2	0.2	1–5 h
3.	Duodenum	0.3	0.02	5 min
4.	Jejunum	3	100	1–2 h
5.	Ileum	4	100	2–3 h
6.	Colon	1.5	3	15–48

**Table 2:** Marketed formulations with innovators who have successfully diminished fast-fed variability.

S. NO.	Branded Name	Drug	Formulation	Manufacturer
1.	Prograf^®^	Tacrolimus	Amorphous solid dispersion	Astellas Pharma US, Inc., Northbrook, IL, USA
2.	Kaletra^®^	Ritonavir/lopinavir	Amorphous solid dispersion	AbbVie Inc., North Chicago, IL, USA
3.	Zortress^®^/Certican^®^	Everolimus	Amorphous solid dispersion	Novartis Pharmaceuticals Corporation East Hanover, NJ, USA
4.	Zelboraf^®^	Vemurafenib	Amorphous solid dispersion	Genentech, Inc., South San Francisco, CA, USA
5.	Ceftin^®^	Cefuroxime axetil	Amorphous form of drug	GlaxoSmithKline Inc., Collegeville, PA, USA
6.	Accupril^®^	Quinapril HCl	Amorphous form of drug	Pfizer Inc., New York, NY, USA
7.	Crestor^®^	Rosuvastatin Calcium	Amorphous form of drug	AstraZeneca Pharmaceuticals LP, Wilmington, DE, USA
8.	Zepatier^®^	Elbasvir/Grazoprevir	Amorphous form of drug	Merck & Co., Inc., Rahway, NJ, USA
9.	Agenerase^®^	Amprenavir	Lipid based formulation	GlaxoSmithKline Inc., Collegeville, PA, USA
10.	Avodart^®^	Dutasteride	Lipid based formulation	GlaxoSmithKline Inc., Collegeville, PA, USA
11.	Procardia^®^	Nifedipine	Lipid based formulation	Pfizer Inc., New York, NY, USA
12.	Rapamune^®^	Sirolimus	Lipid based formulation	Pfizer Inc., New York, NY, USA
13.	Amitiza^®^	Lubiprostone	Lipid based formulation	Sucampo Pharma Americas LLC, Bedminster, NJ, USA and Takeda Pharmaceuticals U.S.A., Inc., Lexington, MA, USA
14.	Hycamtin^®^	Topotecan HCl	Lipid based formulation	Novartis Pharmaceuticals Corporation East Hanover, NJ, USA
15.	Akynzeo^®^	Netupitant	Lipid based formulation	Helsinn Therapeutics (U.S.), Inc. Iselin, NJ, USA
16.	Prometrium^®^	Progesterone	Lipid based formulation	Virtus Pharmaceuticals, LLC, Langhorne, PA, USA
17.	Absorica^®^	Isotretinoin	Lipid based formulation	Sun Pharmaceutical Industries, Inc., Princeton, NJ, USA
18.	Zemplar^®^	Paricalcitol	Lipid based formulation	AbbVie Inc. North Chicago, IL, USA
19.	Vyndaqel^®^	Tafamidismeglumine	Lipid based formulation	Pfizer Inc., New York, NY, USA
20.	Xtandi^®^	Enzalutamide	Lipid based formulation	Astellas Pharma US, Inc. Northbrook, IL, USA
21.	Lipantil Supra^®^	Fenofibrate	Nanocrystal	AbbVie Inc. North Chicago, IL, USA
22.	Emend^®^	Aprepitant	Nanocrystal	Merck & Co., Inc., Rahway, NJ, USA
23.	Triglide^®^	Fenofibrate	Nanocrystal	Skye Pharma Inc., San Diego, CA, USA
24.	Rapamune^®^	Sirolimus	Nanocrystal	Pfizer Inc., New York, NY, USA
25.	Sporanox^®^	Itraconazole	Cyclodextrin	Janssen Pharmaceuticals, Inc. Titusville, NJ, USA
26.	Lynparza^®^ (capsule)	Olaparib	Crystalline solid dispersion	AstraZeneca Pharmaceuticals LP, Wilmington, DE, USA and Merck & Co., Inc., Rahway, NJ, USA
27.	Lynparza^®^ (tablet)	Olaparib	Hot-melt extrusion followed by compression of crystalline solid dispersion	AstraZeneca Pharmaceuticals LP, Wilmington, DE, USA and Merck & Co., Inc., Rahway, NJ, USA

**Table 3:** Formulation approaches for reducing fast-fed state variability with pharmacokinetic data.

Formulation Approaches	Drug	Fasted State		Fed State		Refs
		AUC_0–∞_ (ng.h/mL)	C_max_ (ng/mL)	AUC_0–∞_ (ng.h/mL)	C_max_ (ng/mL)	
Prodrug approach	Enalapril	1209 ± 203	154 ± 39	1173 ±212	147 ± 36	[96]
Cyclodextrin complexation	Amiodarone HCl	1788 ± 121	3.024 ± 0.6631	1911 ± 141	3.314 ± 0.6139	[104]
Osmotic delivery system	Methylphenidate HCl	1857 ± 224	112.6 ± 15.6	1872 ± 242	124.9 ± 17.9	[107]
Solid dispersion	Ziprasidone HCl	874.265 ± 3.908	122.116 ± 2.081	988.67 ±4.234	123.457 ± 1.987	[116]
Nanocrystal technology	Lurasidone HCl	4718.81 ± 638.37	353.72 ± 21.83	4796.30 ± 562.44	360.70 ± 20.71	[10]
SNEDDS	Cinnarizine	1386 ± 474	372 ± 101	1961 ± 324	389 ± 57.0	[145]

**Table 4:** Potential molecular factors contributing to fast-fed variability in terms of AUC and C_max_ of marketed drugs for which products have been developed claiming to reduce the food effect. Data obtained from FDA Drug Label database and European Summary of Pharmaceutical Characteristics (SPC).

S. NO.	Marketed Drugs with High Fast-Fed Variability	pH-Dependent Solubility	pK_a_	Partition Coefficient	Molecular Weight	BCS Class	AUC_fed_/AUC_fast_	C_maxfed_/C_maxfast_
1.	Tacrolimus	Acidic	9.96	3.19	804.08	II	0.63	0.23
2.	Ritonavir	Acidic	13.68	3.9	720.946	II	0.79	0.78
3.	Everolimus	Acidic	9.96	7.4	958.224	III	0.84	0.40
4.	Vemurafenib	No	7.1	4.62	489.92	IV	4.6	2.5
5.	Cefuroxime axetil	Acidic	10.92	0.89	510.475	II	1.41	1.43
6.	Quinapril HCl	Acidic	5.2	1.96	438.516	II	0.75	-
7.	Rosuvastatin Calcium	Basic	4.6	1.92	1001.14	II	1	0.8
8.	Elbasvir/Grazoprevir	Basic	3.77	3.34	882.05	II	1.5	2.8
9.	Amprenavir	Acidic	13.61	2.2	505.628	II	0.79	0.64
10.	Dutasteride	Acidic	12.56	6.8	528.53	II	-	0.85
11.	Nifedipine	No	3.93	2.5	346.335	II	1	0.74
12.	Sirolimus	Acidic	9.96	4.85	914.172	II	1.35	-
13.	Lubiprostone	No	4.3	2.76	390.462	II	1	0.45
14.	Topotecan HCl	Acidic	10.50	−0.88	457.9	IV	1	1
15.	Netupitant	Acidic	9	7.26	578.59	II	1.1	1.2
16.	Progesterone	Acidic	18.92	3.87	314.46	II	1.99	5.19
17.	Isotretinoin	Basic	5	6.3	300.44	II	1.5	1.26
18.	Paricalcitol	No	14.81	4.5	416.36	III	1	1
19.	Tafamidismeglumine	Basic	3.6	4.21	503.33	IV	1	1
20.	Fenofibrate	Basic	3.1	5.24	360.831	II	1.58	-
21.	Aprepitant	Acidic	9.7	4.8	534.427	IV	1.4	-
22.	Itraconazole	Acidic	3.7	5.56	705.64	II	0.76	0.42
23.	Olaparib	No	12.07	1.49	435.08	IV	1.2	1

**Table 5:** Patents filed exclusively to reduce fast-fed variability.

S. NO.	Patent	Title	Formulation Approach Used	Refs
1.	US20110311594A1	Controlled release compositions with reduced food effect.	Bilayered controlled release.	[154]
2.	US20140212491A1	Combination formulation of two antiviral compounds.	Solid dispersion.	[155]
3.	CN103211759B	Puerarin nanocrystalline medical composition and preparation method thereof.	Nanocrystal.	[156]
4.	CN102497857A	Nanostructured sildenafil base, its pharmaceutically acceptable salts and cocrystals, compositions of them, process for the preparation thereof, and pharmaceutical compositions containing them.	Cocrystals.	[157]
5.	WO2015145157A1	Pharmaceutical composition comprising pazopanib.	Nanoparticles.	[158]
6.	JP2004523552A	Reduced food intake, fibrates with a fasting effect, the combination of statins.	Microparticles.	[159]
7.	KR101300654B1	Nanoparticulate fibrate formulations.	Nanoparticles.	[160]
8.	US9504652B2	Nanostructured aprepitant compositions, process for the preparation thereof, and pharmaceutical compositions containing them.	Polyvinyl caprolactam-polyvinyl acetate-polyethylene glycol graft copolymer nanoparticles.	[161]
9.	ES2372746T3	Stabilized microparticles fibrate.	Microparticles stabilized by surface active phospholipids.	[162]
10.	US20090028935A1	Carvedilol forms, compositions, and methods of preparation thereof.	Amorphous carvedilol phosphate salt and a complexing agent and controlled release of amorphous form.	[163]
11.	WO2014009436A1	Nanosuspension of abiraterone acetate.	Nanosuspension of abiraterone acetate.	[164]
12.	CH707330A2	Pharmaceutical compositions with reduced dose of fenofibrate.	A mixture of fenofibrate nanoparticles and micronized fenofibrate.	[165]
13.	US9012511B2	Nanoparticulate cinacalcet compositions.	Cinacacalcet nanoparticles.	[166]
14.	US20080044486A1	Controlled food effect composition.	Membrane lipids for controlled release.	[167]
15.	WO2015145145A1	Pharmaceutical composition comprising lapatinib.	Nanoparticles.	[158]
16.	US20120135053A1	Nanoparticulate telmisartan compositions and process for the preparation thereof.	Nanostructured Telmisartan.	[168]
17.	US20130210794A1	Nanostructured ezetimibe compositions, process for the preparation thereof, and pharmaceutical compositions containing them.	Nanostructured ezetimibe.	[169]
18.	CN101180038A	Nanoparticulate corticosteroid and antihistamine formulations.	Antihistamine corticosteroid nanoparticles.	[170]
19.	KR20080024213A	Nanoparticulate megestrol formulations.	Megesterol acetate nanoparticles.	[171]
20.	JP2012530126A	Nanoparticulate Olmesartan medoxomil composition, method for its preparation, and pharmaceutical composition containing them.	Nano cocrystals.	[172]
21.	ES2302925T3	Nanoparticle compositions, kinase inhibitors, mitogen activated protein (MAP).	Nanoparticles.	[173]
22.	US20090004262A1	Nanoparticulate formulations and methods for the making and use thereof.	Cyclodextrin inclusion complex	[174]
23.	JP2005535582A	Coated tablets.	Phospholipid applied to the surface of the fenofibrate microparticles.	[175]
24.	CN101132768A	Nanoparticulate tacrolimus formulations.	Nanoparticles.	[176]
25.	US20130303495A1	Emulsion formulations.	SNEDDS, SMEDDS, and SEDDS	[177]
26.	US20170112775A1	Situ self-assembling pro-nanoparticle compositions and methods of preparation and use thereof.	Self-assembling pronanoparticles.	[178]
27.	WO2014132134A1	A composition comprising a lipid compound, a triglyceride, and a surfactant, and methods of using the same.	SNEDDS, SMEDDS, and SEDDS	[179]
